# Coronary artery bypass grafting under sole Impella 5.0 support for patients with severely depressed left ventricular function

**DOI:** 10.1007/s10047-021-01285-1

**Published:** 2021-06-24

**Authors:** Shintaro Katahira, Yukiharu Sugimura, Arash Mehdiani, Alexander Assmann, Philipp Rellecke, Igor Tudorache, Udo Boeken, Hug Aubin, Artur Lichtenberg, Payam Akhyari

**Affiliations:** 1 Department of Cardiac Surgery and Research Group for Experimental Surgery, University Hospital and Medical Faculty, Heinrich Heine University Hospital, Moorenstr. 5, 40225 Düsseldorf, Germany; 2 Department of Cardiothoracic Surgery, University Hospital, Tohoku University, Sendai, Japan

**Keywords:** Impella 5.0, OPCAB, Low ejection fraction, Ischemic heart disease, Ischemic cardiomyopathy

## Abstract

Selection of the ideal surgical procedure for coronary revascularization in patients with severe cardiac dysfunction at times may represent a challenge. In recent years, with the advent of surgical large microaxial pumps, e.g., Impella 5.0 (Abiomed Inc., Boston, USA), specific support and effective unloading of the left ventricle has become available. In the interventional field, good results have been achieved with smaller microaxial pumps in the setting of so-called protected percutaneous coronary intervention. In this study, we would like to share our early experience with surgical coronary revascularization under the sole support of Impella 5.0, omitting the use of heart–lung machine in three cases of severe cardiac dysfunction due to complex ischemic heart disease. Effective circulatory support intraoperatively and postoperatively speaks in favor of this technique in selected patients.

**Supplementary Information:**

The online version contains supplementary material available at 10.1007/s10047-021-01285-1.

## Introduction

Growing evidence supports coronary artery bypass grafting (CABG) for ischemic heart disease (IHD) in front of severe cardiac dysfunction [1]. However, operative risk in this particular patient cohort with severely depressed left ventricular (LV) function remains a matter of controversy [2–4].

Off-pump CABG (OPCAB) has been advocated for the advantage of omission of cardiopulmonary bypass (CPB), which by itself is known to trigger perioperative complications, e.g., systemic inflammatory response syndrome and perioperative stroke. Moreover, OPCAB may provide certain advantage over standard CABG because of a lack of cardioplegic arrest and associated myocardial ischemia, potentially aggravating perioperative myocardial damage [2–4]. Here, we report on a surgical method of CABG supported by Impella 5.0 (Abiomed Inc., Boston, USA) and without the use of the heart–lung machine, in the following termed Impella supported coronary artery bypass, ISCAB. We demonstrate the feasibility of this approach by sharing our experience in three successful cases in which Impella 5.0 was inserted preoperatively to stabilize hemodynamics not only during the surgery but also after surgical coronary revascularization without CPB.

## Surgical procedure and perioperative management of Impella 5.0

**Fig. 1:**
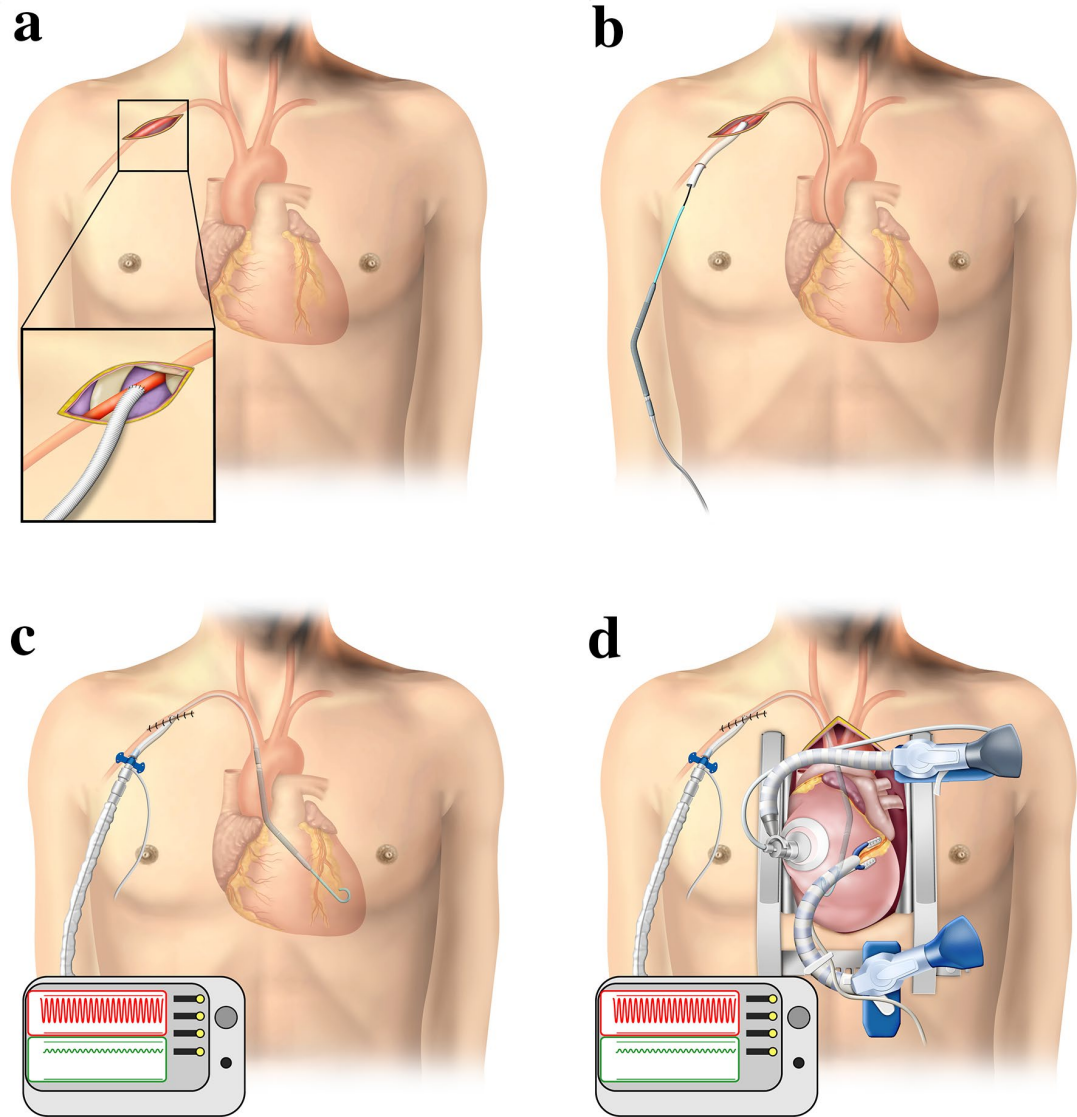
Surgical procedure for off-pump coronary artery bypass grafting with Impella 5.0 support. **a** A skin incision was placed under the right clavicle to secure the right axillary artery. A 10 mm graft was anastomosed by clamping the right subclavian artery. **b**, **c** Impella 5.0 is inserted through the graft which is tunneled subcutaneously about 5 cm away from the infra-clavicular skin incision. **d** The access to the right subclavian artery is closed and Impella 5.0 is put into operation. Standard OPCAB via median sternotomy is performed under the support of Impella 5.0 using standard OPCAB techniques for exposure of the different coronary perfusion territories and for stabilization of the anastomosis region. *OPCAB* off-pump coronary artery bypass grafting

First, insert Impella 5.0 in the operating room prior to sternotomy. The surgical procedure for Impella 5.0 insertion has been described before. In brief, right axillary artery is exposed surgically (Fig. 1a) and a 10 mm graft is anastomosed in end-to-side-fashion after systemic heparinization with a targeted activated clotting time greater than 250 s. Under combined fluoroscopy and trans-esophageal echography (TEE) control and using Seldinger technique Impella catheter is advanced via the anastomosed prosthesis and positioned appropriately (Fig. 1b). Auxiliary flow and LV support are obtained.

Further operation is performed after median sternotomy and under Impella 5.0 support. (Fig. 1c) Revascularization order follows the principles of OPCAB procedure, starting with revascularization of the left anterior descending coronary artery (LAD), continuing with left circumflex coronary artery (LCX) territory and finally addressing the right coronary artery (RCA). When performing revascularization of LCX and RCA, luxation and positioning of the heart is promoted by the use of a stabilizer (Medtronic Octopus, additionally with Medtronic Starfish, if needed; Medtronic, Inc, Minneapolis, MN). At this stage a close monitoring of the position (TEE) and output of Impella 5.0 (displayed by the controller) is mandatory (Fig. 1d). Continuous monitoring of cardiac output and pulmonary artery pressures was performed using pulmonary artery catheter with thermodilution method, as routinely performed at our institution in all OPCAB procedures. For the instance of hemodynamic deterioration, CPB standby was provided for all of herein presented ISCAB procedures. Moreover, intraoperative cell saver is used to support intraoperative patient blood management.

The end of the operation, Impella 5.0 support is continued for the early postoperative period, including extubation and weaning from inotropes as well as vasopressors. Intermittent evaluation by transthoracic echocardiography (TTE) or TEE serves as a crucial guiding factor to assess heart function and to decide on the optimal time point for Impella 5.0 explantation.

## Case presentation

**Fig. 2:**
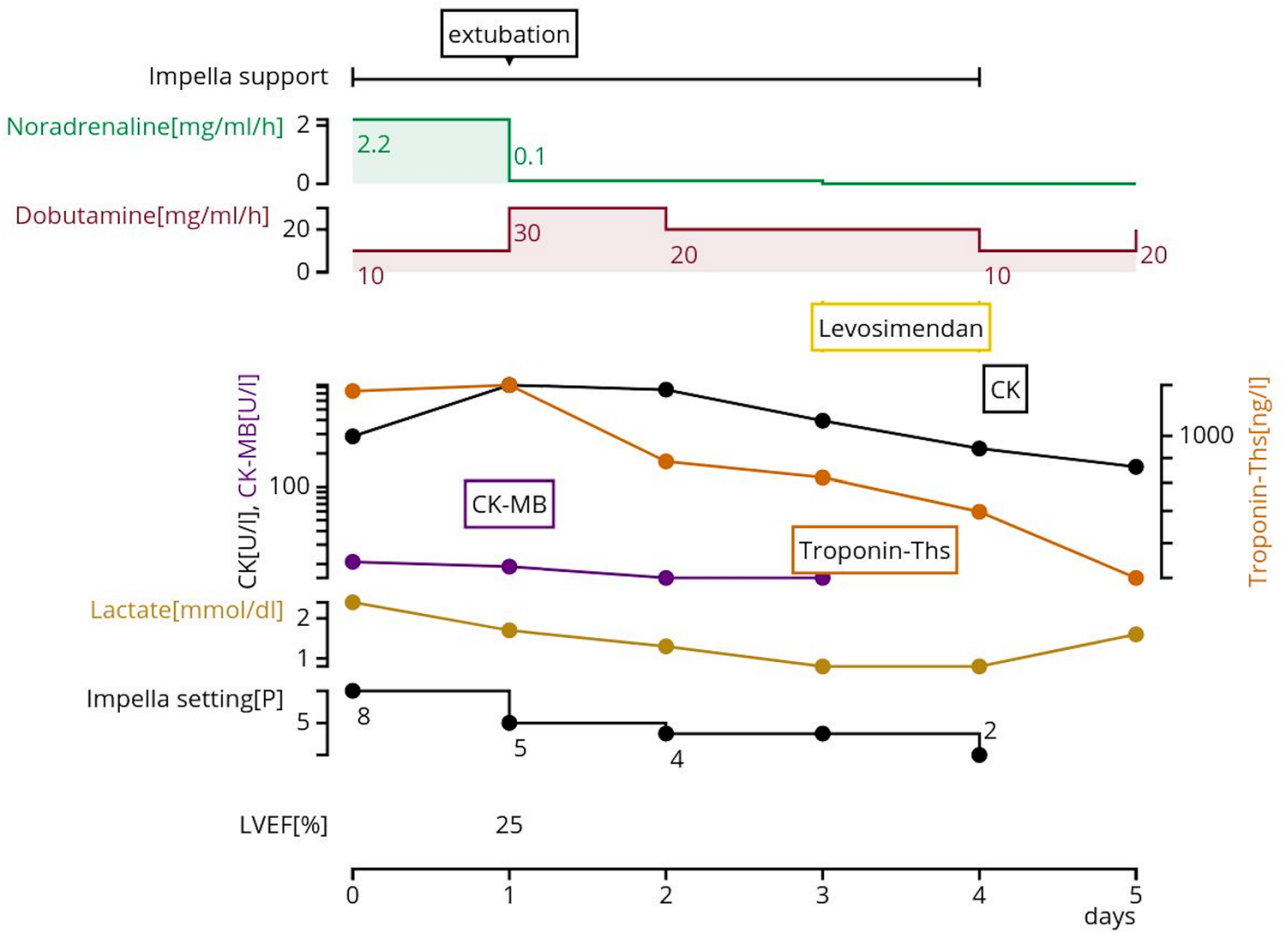
Postoperative hemodynamic profile and the recovery of heart enzymes of Case 2. *CK* creatinine kinase, *LVEF* left ventricular ejection fraction, *Troponin Ths* troponin T high sensitive

All three consecutive patients presented here fully recovered after surgery and were discharged on POD 27.0 ± 3.46 without major adverse cardiovascular events. The total operation time was 388.3 ± 39.5 min, with an average of three bypasses. Table 1 shows key points of the course in these three patients. Further, the postoperative hemodynamic profile and the recovery of heart enzymes of Case 2 is shown in Fig. 2.

**Table 1:** Three cases overview

	Case		
	1	2	3
Patients characteristics			
Age (years)	58	52	65
Sex	M	M	M
Arterial hypertension	+	+	+
Hyperlipidemia	+	−	−
Diabetes	−	+	−
History of PCI	−	+	−
NYHA	4	3	3
Emergent/urgent	Emergent	Urgent	Urgent
Indication for CABG	3VD	3VD	LMT + 3VD
Operation time (min)	353	381	431
Preoperative EF (%)	20	20	25
Postoperative EF (%)	30	30	29
Preoperative LVEDD (mm)	70	54	62
Postoperative LVEDD (mm)	58	55	62
Preoperative RVEF (%)	N/A	55	60
Preoperative RVEDD (mm)	N/A	30	30
Preoperative TAPSE (mm)	17.2	18.5	19.5
Preoperative TR	Trivial	1	Trivial
Impella 5.0 support duration (days)	10	4	11

*M* male, *PCI* percutaneous coronary intervention, *NYHA* New York Heart Association, *CABG* coronary artery bypass grafting, *3VD* three vessel disease, *LMT* left main trunk, *EF* ejection fraction, *LVEDD* left ventricular end-diastolic diameter, *RVEF* right ventricular ejection fraction, *RVEDD* right ventricular end-diastolic anteroposterior diameter, *TAPSE* tricuspid annular plane systolic excursion, *TI* tricuspid regurgitation

### Case 1

A 58-year-old man was diagnosed with non-ST segment elevation myocardial infarction (NSTEMI) complicated by cardiogenic shock (CS) and was transferred to our hospital by ambulance. Coronary angiography (CAG) showed severe coronary stenosis in all three territories. TTE demonstrated severely reduced LV ejection fraction (EF) of 20% and LV end-diastolic diameter (LVEDD) of 70 mm. Impella 5.0 was inserted and coronary revascularization with four bypasses in ISCAB technique was performed: left internal thoracic artery (LITA) to LAD, saphenous vein graft (SVG) to the posterior descending branch (PD), sequential SVG to intermediate branch (IM) and further to obtuse marginal branch (OM). Postoperative course was complicated by re-thoracotomy because of postoperative pericardial tamponade. No active bleeding site was observed after re-thoracotomy. After explantation of Impella 5.0 on 10th postoperative day (POD) the patient was transferred to cardiac rehabilitation on 31st POD.

### Case 2

A 52-year-old man was admitted on emergency basis and STEMI was confirmed. Emergency percutaneous coronary intervention (PCI) for RCA as culprit lesion was performed. 5 days later, he was introduced to our department for the purpose of left coronary artery revascularization. Severely decreased LVEF (20%) was revealed by TTE. Since meanwhile he was not in CS, urgent revascularization was scheduled following the principles of ISCAB (LIMA-LAD, SV-OM) combined with sole Impella 5.0 support. Impella 5.0 was removed on 4th POD. The patient had a good progress and was discharged from the hospital on 25th POD.

### Case 3

A 65-year-old man was admitted to another hospital because of acute heart failure (HF). CAG revealed severe three vessel disease including left main trunk lesions. After stabilization of HF, he was referred to our department for the purpose of surgery in NYHA class III. Preoperative TTE showed significantly dilated LV (LVEDD: 62 mm) with severely depressed LVEF of 25% with global hypokinesis. Urgent revascularization was performed (LIMA-LAD, SVG-OM, SVG-PD) following ISCAB technique under sole Impella 5.0 support. Postoperative course was complicated by viral pneumonia, however, Impella 5.0 was successfully weaned on 11th POD and he was transferred for rehabilitation on 25th POD.

## Discussion

Impella 5.0 is indicated for patients with circulatory failure refractory to conservative therapy options [5–7]. Most recent literature in the field of HF management suggests an increasing use of large microaxial pumps as bridging therapy or as a temporary perioperative or peri-procedural support. Impella 5.0 is able to effectively unload LV, thereby reducing wall stress and myocardial oxygen consumption [8]. In the setting of low cardiac function or CS good results have been obtained for Impella supported PCI [9]. However, the value of perioperative Impella support for high-risk CABG in patients with severely reduced LV function is yet to be determined.

Current evidence speaks in favor of CABG for IHD with LV dysfunction, but the appropriate operative method is still controversial [1]. A recent comparison between on-pump CABG and OPCAB for coronary revascularization in patients with LV dysfunction has demonstrated a reduction of postoperative complications by OPCAB [3]. On the other hand, in randomized controlled trials OPCAB has been associated with lower numbers of grafts and incomplete revascularization, which may have a negative effect on long-term results of surgical revascularization [4]. In present series, despite severely impaired LV function complete revascularization has become feasible after stabilization of hemodynamics by implantation of Impella 5.0 prior to surgical revascularization on beating heart without the use of CPB. As this procedure employs a microaxial blood pump but yet avoids the use of CPB, we termed this procedure Impella supported CAB (ISCAB). In addition, Impella 5.0 was able to operate without any problem even during luxation of the heart for revascularization of the LCX or RCA perfusion territory. Since LV was unloaded, the surgical field for anastomosis could be secured, providing certainly a wider range of freedom than in comparable cases with LV dilatation and reduced EF. Although the LCX anastomosis was performed in a stable hemodynamics, the precise performance and variations in circulatory support by Impella 5.0 at the time of elevating apex remains yet to be evaluated in larger patients’ cohorts. Further, the possibility of injury to cardiac structures during maneuvers of cardiac positioning for better exposure of the LCX and RCA perfusion territory warrants further evaluation of this technique, meanwhile it is considered necessary to perform any elevation maneuvers carefully and under repetitive TEE-control of the position of Impella tip [10].

Another advantage of using Impella 5.0 lies in its usefulness as a ventricular support device not only in the pre- and intra-operative time, but also in the early postoperative period. IABP (Intra-Aortic Balloon Pumping) and veno-arterial extracorporeal membrane oxygenation (VA-ECMO) have also been used for temporary support after CABG in high-risk patients, however, both systems have shown considerable limitations when used in patients with severe LV dysfunction, due to problems with the limited maximal assist flow rate or due to detrimental increase in LV afterload, respectively [5–7]. In contrast, Impella 5.0 is highly effective in unloading LV while supporting peripheral circulation and preventing organ failure caused by low output syndrome [11]. In fact, in our case series, despite the preoperatively severe cardiac dysfunction, all patients recovered without deterioration of organ function.

Additionally, in the case of such severely impaired LV function, on-pump beating CABG with CPB may present an alternative. However, in that case, VA-ECMO, IABP or both may become necessary after surgery. Therefore, and also from an economic point of view, it was considered better to use only Impella 5.0.

We were able to obtain good results by performing ISCAB, i.e., CAB under Impella 5.0 support, for IHD with severe cardiac dysfunction. Some previous articles support our favorable result of ISCAB for patients with severely impaired LVEF [12, 13]. With sufficient monitoring when elevating the heart apex, this strategy is considered to be an option for patients with severe cardiac dysfunction to perform facilitated surgical revascularization with omission of heart–lung machine and protected hemodynamic stability in the perioperative course.

## Supplementary Information

Below is the link to the electronic supplementary material.

Supplementary file1 (TIFF 73248 KB) Supplementary Material Postoperative hemodynamic profile and the recovery of heart enzymes of 1; Case 1, 2; Case 3. CK; creatinine kinase, LVEF; left ventricular ejection fraction, Troponin Ths; troponin T high sensitive

Supplementary file2 (TIFF 73248 KB)
